# The Application of Computer-Based Multimedia Technology in Cognitive Computing

**DOI:** 10.1155/2022/3354576

**Published:** 2022-02-26

**Authors:** Yu Wang

**Affiliations:** Modern Educational Technology Center, Qiqihar Medical University, Qiqihar, Heilongjiang 161006, China

## Abstract

With the continuous development of science and technology, people's research on computer-related technologies is gradually deepening. The proposal of artificial intelligence makes the research of intelligent AI in urgent need for seeking breakthroughs. Among them, cognitive computing methods are important for computers and human brain thinking. Model learning is even more meaningful. This article aims to study the application of computer-based multimedia technology in cognitive computing methods. For this, this article proposes a method of distributed multimedia technology and a method of two-way cognition for cognitive computing through this technology to deepen the rapid improvement of cognition. In addition, this article finally designed relating experiments to study it. The experimental results show that the cognitive accuracy of the improved cognitive computing method has increased by 32.9%, and the cognitive ability has also been greatly improved compared to the past.

## 1. Introduction

In the era of unlimited development of digital technology, people's cognitive processes and cognitive actions are increasingly dependent on the development of technology. Imagine that modern people rely on mobile phones, iPhone, laptops, and the Internet. If they lose those external devices, what will happen to cognitive action? Scientists leave the laboratory to understand nature through machines. As more and more cognitive experiences are carried out in the form of interaction between humans and computers, then where is the human heart? These questions prompt people to start thinking about what human cognition is, what is the subject of cognition, what is the heart, where is the heart, the relationship between the heart and the outside world, and other formal problems of modern cognitive technology. Can technology constitute the basic elements' form of an extension? The transformation from the external auxiliary media of cognitive technology to the internal composition of the cognitive process reflects the profound influence of technology, which is extended form and their interaction.

Comparing with the previous cognitive technology, modern cognitive technology emphasizes the cognitive function of the human brain and the integrated technology of externalization. When these technologies are applied to our cognitive practice, they also accompany our cognitive process. When they are helpful to our cognition, they actually have a great impact on the nature of our thoughts and cognition. On the one hand, technology has gradually changed from traditional tools, media, and auxiliary functions to more and more important cognitive components and can even determine the appearance of cognitive objects. On the other hand, modern cognitive technology is very important for simulating human perception and strengthening human cognitive expansion. People's cognition is very important.

Modern cognitive technology has gained people's attention as the direction of independent research in the technical field from the beginning. Just like the law of evolution shown in technology ecology, modern cognitive technology is the focus of relating technologies after facing research difficulties. For example, Coccoli et al. investigated how the rise of big data and cognitive computing systems will redesign the labor market and also affect the learning process. In this regard, they referred to higher education and described the model of the smart university, which relied on the concept as the basis for the development of new smart cities. Therefore, they regards education as a process so that we can find specific problems to solve existing criticisms and make some suggestions on how to improve the university performance [1]. Roy et al. proposed that random switching of devices can also be used for applications involving deep belief networks and Bayesian inference. They explained the probabilistic intelligent computing unit system from a multidisciplinary perspective [2]. Memeti and Pllana use advanced parallel programming technology to study the available resources in parallel computing systems. They summarized the mistakes often made by programmers and introduced the Parallel Programming Assistant (PAPA) to everyone. The effect is very obvious [3]. Dessl et al. propose that moving towards the next generation of personalized learning environments requires intelligent methods supported by analysis for advanced learning environments with rich digital content. However, as the number of videos continues to increase, it becomes challenging to arrange and search according to specific categories. So, they solved this problem by bypassing the traditional terminology-based method [4]. Demirkan et al. proposed that cognitive computing refers to large-scale learning, purposeful reasoning, and intelligent systems that naturally interact with humans and other intelligent systems [5]. Li et al. designed a cognitive computing framework in which legal factors are first expressed in a formal way; secondly, legal factors are extracted, combined with rules-based and deep learning methods, to expand concepts and their relationships. After applying the induction rules, their machine learning prediction results are easily understood by the public [6]. Kaur et al. discussed the important role that cognitive computing can play in an organizational environment with complex relationships. Their research highlights the main gaps in traditional decision-making systems. As a facilitator of smart positioning and a facilitator of information access, cognitive computing improves work efficiency [7]. Zhu et al. proposed that with the development of computing and interactive systems inspired by cognition, cognition is becoming a new and promising methodology, which makes a large number of applications possible and is of great significance to changing our lives. How to apply machine learning, intelligent interaction, and cognitive science to APP design to improve human cognitive ability is worthy of deep consideration and exploration [8]. The abovementioned documents describe the technical points of the key technologies involved are very accurate, and the depth of research on some technologies is also involved to a considerable extent. They are a good reference for the research topics of this article, but for some technology feasibility, this article did not go to an experimental design to verify, resulting in the credibility of the literature being not particularly high.

The innovation of this article is to understand the cognitive model of cognitive computing in detail through the separation of cognitive computing and propose a two-way cognition method. And the key multimedia technology has also been improved, and the distributed multimedia technology has been cleverly adapted to ensure the feasibility of the article research. The conceptual cognitive ability of improved cognitive computing is also explored in the experiment and analysis part, and its specific effects are also explained.

## 2. Cognitive Computing for Multimedia Applications

### 2.1. Cognitive Computing That Mimics the Working Mechanism of the Human Brain

When human beings deal with daily things, they are often in a complex and changeable environment, which results in a very complicated amount of information input into the brain. There are vision, hearing, touch, smell, and so on; all kinds of information enter the brain through their own signals. In order to ensure the efficiency of processing, the brain does not process most of the useless information, but concentrates on the processing and analysis of a part of the useful information. This is the mechanism of attention selection and adjustment [9]. Experimental studies have shown that in the case of recognizing and not recognizing specific signals, because the neuron strength of the prefrontal cortex neurons is completely different, the prefrontal cortex neurons play an important role in selecting and regulating attention. Since the structure and function of neurons in the prefrontal cortex are higher than those of other cortexes such as the sensory and motor cortexes, the mechanism of this selection and regulation is top-down. In other words, neurons in the prefrontal cortex apply information to other cortexes that respond to the focus of relevant precautions to execute the current event. The prefrontal cortex works with the medial temporal lobe and the cerebral cortex to record individual experiences with time stamps to form episodic memory, which plays an important role in the formation and recall of episodic memory. And the selected result information will be further analyzed and processed, including the learning process.

**Figure 1:**
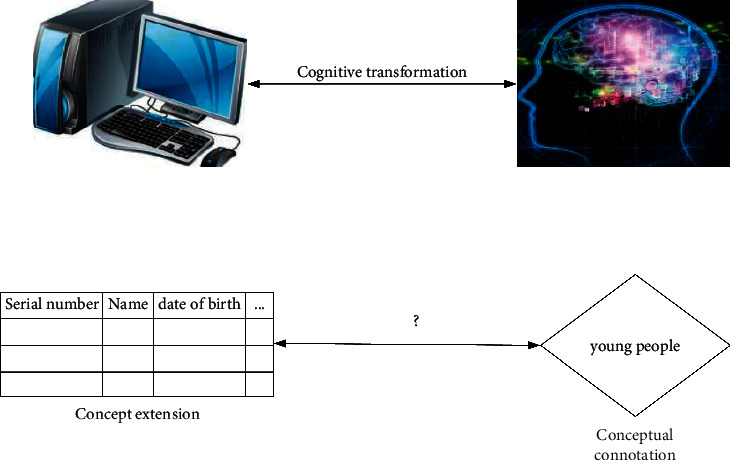
Two-way cognitive transformation between concept connotation and denotation.

A concept is a high-level product of the human brain, and it is the reflection of an object in the human brain. Concepts may be subjective, but in essence they are some sort of classification rules that reflect the general or most important attributes of things. People use concepts to abstract and categorize various objects in the real world according to their essential characteristics and construct cognitive structures that are not too complex to measure with psychological scales. Natural language is a tool of human thinking (the cognition rises from a low-level perceptual stage to a high-level rational stage. The process of forming this concept in the human brain is a manifestation of thinking) and has an important position in artificial intelligence. It expresses concepts through language values, and these concepts are usually uncertain. In dealing with uncertainty, there have been some theoretical models, such as probability theory dealing with random uncertainty and fuzzy sets and rough sets dealing with fuzzy uncertainties. But in studying the randomness and ambiguity of natural language, these theories fail to combine the two well. The cloud model studies the uncertainty of natural language from the perspective of fuzziness and randomness and uses digital features to express the connotation of the concept as a whole. It realizes the mutual cognitive conversion between the connotation and extension of the concept through cloud transformation (CT) [10].  Figure 1 shows its conversion process.

**Figure 2:**
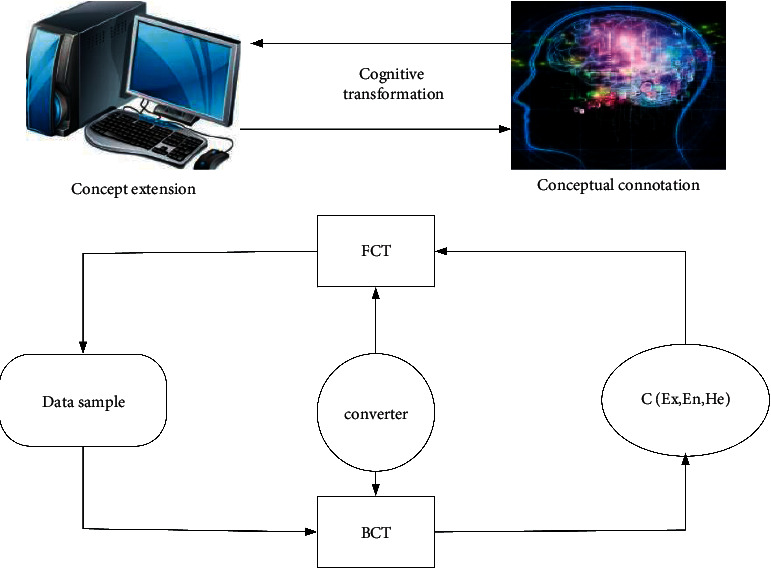
Schematic diagram of the two-way cognitive computing process based on CT.

In the cloud model, forward cloud transformation is to convert a qualitative concept (conceptual connotation) expressed by digital features into its quantitative representation (concept extension), and inverse cloud transformation is to convert some quantitative data (concept extension) into a qualitative concept represented by digital features (conceptual connotation). The forward CT and the inverse CT are used for multiple cycles in both directions; that is, a qualitative concept (intention) is generated through the forward CT algorithm to generate quantitative data (extension), and then the inverse CT algorithm is used to form a qualitative concept (intention). This cycle is repeated many times to simulate the two-way cognitive computing process of humans on the concept [11], as shown in Figure 2.

**Figure 3:**
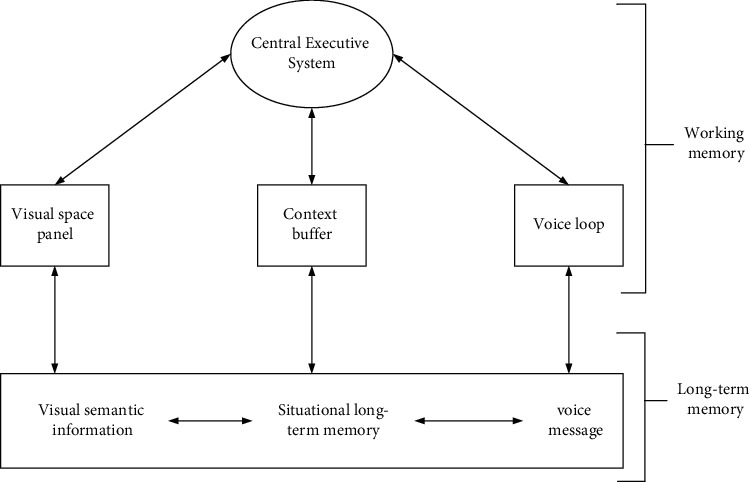
Memory system model.

Therefore, according to the characteristics of feeding forward CT and inverse CT, computer algorithms can be used to simulate and realize the two-way correlation calculation process of people's concepts [12, 13]. In principle, long-term memory preserves the personal factual knowledge of the outside world such as objects and scenes, as well as the formal knowledge of the preservation actions, actions, and operations of the long-term memory in the process. In the case of long-term memory, save personal experience knowledge and experience activities. Short-term memory is based on the needs of work, using specific methods to call long-term memory knowledge, and the knowledge emphasized in short-term memory will also be stored in long-term memory [14]. Until a new subsystem—Scenario Buffer—was proposed, how different types of information are integrated and processed is analyzed and discussed. This latest working memory model has been widely recognized by all walks of life. The schematic diagram of the model is shown in Figure 3.

According to the functional description of the prefrontal cortex and hippocampus structure, as well as the working mechanism of the hippocampus-prefrontal neural circuit, working memory is designed [15]. At the same time, learn from the memory system model, design long-term memory, work in coordination with working memory, and control the learning process of the model. This structure composed of working memory and long-term memory is called the hippocampus-prefrontal memory system, which is the core part of the model proposed in this article [16].

**Figure 4:**
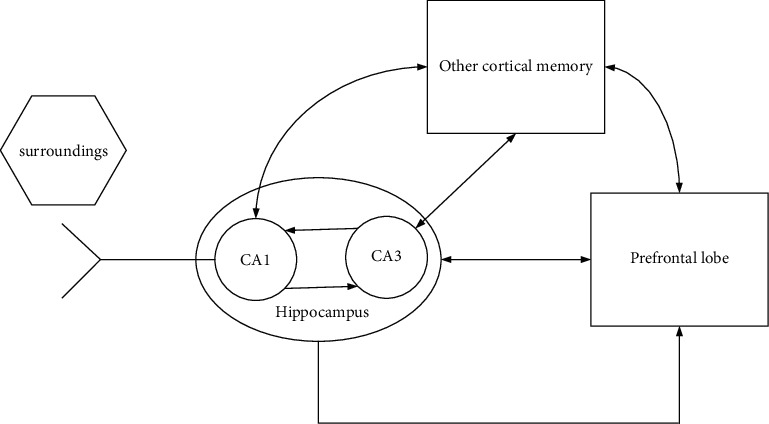
The brain hippocampus-prefrontal neural circuit and related memory system model after transformation.

According to the description and analysis of the hippocampus, prefrontal lobe, and memory system models, the memory system with the hippocampus-prefrontal neural circuit as the core is simplified. The simplified hippocampus-prefrontal memory system is shown in Figure 4.

Among them, the hippocampus is mainly composed of the CA1 subregion, which generates internal motivation of visual strangeness, and the CA3 subregion, which activates memory communication and storage. The hippocampus and the prefrontal lobe and other cortical memories have extensive interactions and connections and jointly complete high-level cognitive functions [17].

### 2.2. Two-Way Cognitive Model

A concept is a high-level product produced by the human brain after processing external information, and it is the qualitative expression of things in the human brain. And this kind of expression is personally subjective, but it is essentially the same, reflecting the general law of things or the most important attribute that can express its connotation. Only concepts are stored in the human brain. All things in the world can be abstractly classified according to their unique characteristics, forming one or several relatively simple concepts and storing them in the human brain. This process is the process in which human cognition rises from the low-level perceptual stage to the high-level rational stage. At its root, the process of concept formation is the process of cognition, that is, the process from perceptual to rational. The uncertainty of things leads to uncertainty in the cognitive process [18].

The purpose of the cloud model is to study the uncertainty of the concept and to realize the mutual conversion between the connotation and extension of the concept through cloud transformation. And the cognitive computing model simulated by the two-way cloud model has more important significance in exploring the essence of human cognition. The cloud model is defined as follows.

Set *U* as a quantitative domain with precise values, *C* as a qualitative concept, and *μ* ∈ [0, 1] as a stable random number.

(1) $$\begin{array}{c} \mu :U⟶\left[0,1\right], \end{array}$$

(2) $$\begin{array}{c} \forall x\in U,x⟶\mu \left(x\right). \end{array}$$

Each *x* is called a cloud drop in the universe of discourse.

The definition of the cloud model is the same as above, if *x* satisfies

(3) $$\begin{array}{c} x=R\left(\text{Ex}|y\right). \end{array}$$

Consider

(4) $$\begin{array}{c} y=R_{n}\left(\text{En},\text{He}\right). \end{array}$$

And the certainty of *x* to *C* is

(5) $$\begin{array}{c} u\left(x\right)=e^{-{\left(x-E_{x}\right)}^{2}/2y^{2}}. \end{array}$$

In the forward transformation of the second-order normal cloud algorithm, *n* cloud drops are generated through the four digital features of expected value Ex, entropy En, super-entropy He, and cloud drop number *n* so as to generate cloud clusters that can reflect the conceptual connotation [19].

Let *x*_*i*_ be a cloud drop element of the concept. For this element, the certainty of the value can be calculated by the following formula, which is

(6) $$\begin{array}{c} u\left(x\right)=e^{\left(-{\left(x-E_{x}\right)}^{2}/2y^{2}\right)}. \end{array}$$

For the contribution of the second-order normal cloud drop, the contribution of the cloud drop to the qualitative concept can be obtained by the following formula, which is

(7) $$\begin{array}{c} \Delta C\approx \frac{\mu_{A}\left(x\right)\cdot \Delta x}{\sqrt{2\pi }\text{En}}, \end{array}$$

where ∆*x* is the cloud drop group and ∆C is the contribution of ∆x to the qualitative concept. From the above formula, it can be concluded that the total contribution of all cloud drops in the universe to the qualitative concept is 1, which is

(8) $$\begin{array}{c} C=\frac{\int_{-\infty }^{+\infty }\mu_{A}\left(X\right)\text{d}x}{\sqrt{2\pi }\text{En}}, \end{array}$$

(9) $$\begin{array}{c} \frac{\int_{-\infty }^{+\infty }e^{-{\left(x-Ex\right)}^{2}/2{\text{En}}^{2}}}{\sqrt{2\pi }\text{En}}=1. \end{array}$$

The cloud model algorithm uses normal random numbers to generate cloud drops, and the generation process of cloud drops conforms to probability theory. The generation of cloud drops once is the condition for another generation, and the second-order normal cloud drops are as follows:

(10) $$\begin{array}{c} X_{i}=R_{N}\left(E_{X},\left|y_{i}\right|\right), \end{array}$$

(11) $$\begin{array}{c} y=R_{N}\left(\text{En},\text{He}\right). \end{array}$$

Therefore, the certainty of *x* to *C* is

(12) $$\begin{array}{c} u\left(x\right)=e^{-{\left(x-E_{x}\right)}^{2}/2y^{2}}. \end{array}$$

Since *y* = *R*_N_(En, He), the random variable *Y* obeys the normal distribution with En as the expectation and He as the standard deviation, so the probability density function of *Y* is

(13) $$\begin{array}{c} f_{y}\left(y\right)=\frac{e^{-{\left(y-E_{N}\right)}^{2}/2He^{2}}}{\sqrt{2\pi }He}. \end{array}$$

When the random variable *Y* = *y* is a fixed value, since *x* = *R*_N_(*Ex*, |*y*|), the probability density function of the random variable *X* is

(14) $$\begin{array}{c} f_{x/y}\left(x|Y=y\right)=\frac{e^{-{\left(x-E_{n}\right)}^{2}/2He^{2}}}{\sqrt{2\pi }\left|y\right|}. \end{array}$$

The conditional probability density formula is

(15) $$\begin{array}{c} f_{x,y}\left(x|y\right)=f_{x/y}\left(x|Y=y\right)f_{Y}y. \end{array}$$

From the above two formulas, the probability density function of random variable *X* can be obtained as

(16) $$\begin{array}{c} f_{x}\left(x\right)=\int_{-\infty }^{+\infty }f_{x,y}\left(x,y\right)\text{d}y=\frac{1}{\sqrt{2\pi }\text{He}}\int_{-\infty }^{+\infty }\frac{1}{\left|y\right|}e^{-{\left(x-E_{X}\right)}^{2}/2y^{2}-{\left(y-E_{N}\right)}^{2}/2{\text{He}}^{2}}\text{d}y. \end{array}$$

When He is 0, the probability density function of random variables is the probability density function of N(Ex, He) [20]. For the second-order normal cloud, it has the following mathematical properties:

*X*'s mathematical expectation:

(17) $$\begin{array}{c} \text{EX}=\text{Ex}. \end{array}$$

Variance of *X* is

(18) $$\begin{array}{c} \text{DX}={\text{En}}^{2}+{\text{He}}^{2}. \end{array}$$

The third central moment of *X* is

(19) $$\begin{array}{c} E{\left(X-\text{EX}\right)}^{3}=0 \end{array}$$

*X*'s fourth-order central moment is

(20) $$\begin{array}{c} E{\left(X-\text{EX}\right)}^{4}=9{\text{He}}^{4}+18{\text{En}}^{2}{\text{He}}^{2}+3{\text{En}}^{4}. \end{array}$$

Among them, He characterizes the cloud fogging level.

When *Y* = *y* is a fixed value, the probability density function of the random variable *z* with the degree of certainty *u*(*x*) is

(21) $$\begin{array}{c} f_{Z/Y}\left(z|Y=y\right)=\left\{\begin{array}{c} \frac{1}{\sqrt{-\pi \;\ln\;z}},0<z<1\\ 0, \end{array}\right.. \end{array}$$

It can be seen from the above formula that the probability density function of the random variable *z* of *u*(*x*) has nothing to do with *Y*. This shows that the laws reflected in the process of people's cognition of various concepts are indistinguishable, so this method is actually effective.

### 2.3. Multimedia Technology

The reason why multimedia technology is so significant to the field of cognitive computing is that multimedia technology itself has many features and functions that other media such as slideshows, projections, movies, sound recordings, video recordings, and television do not have or are not fully equipped. Especially because multimedia has the characteristics of pictures, text, sound, and even moving images, it can provide the most ideal cognitive computing environment, and it will inevitably have a profound impact on the development of cognitive computing. The visualization and interactivity of its combination with computer technology enables learners to learn actively and creatively [21]. The multimedia technology mentioned here is a computer-centric multimedia technology, which is completely different from the original simple and mechanical combination of various forms of media.

#### 2.3.1. Distributed Multimedia

First of all, “multimedia” in a narrow sense refers to the methods and means (e.g., transmission, storage, processing, etc.) that humans use computers or similar devices to interactively process multimedia information. In a broad sense, “multimedia” refers to information processing a field of all related technologies and methods (including broadcasting and communications, household appliances, and printing and publishing).

Secondly, what is multimedia technology? “Multimedia technology is a technology that integrates text, sound, graphics, still images, dynamic images, etc., with computers.” This was defined by the former chairman of SGI at the meeting. Nowadays, with the development of microelectronics, audiovisual, and computer and communication technology, multimedia technology has become a comprehensive interdisciplinary edge interdisciplinary (computer system structure, hardware technology, software technology, graphics, image processing, animation, sound, signal processing, network, high-speed communication technology, artificial intelligence, and other fields).

With the development of computer and digital communication technology, the meaning of multimedia has been greatly deepened. Distributed multimedia system abbreviated as DMS is more formally defined as follows. A distributed multimedia system is a system that integrates multiple functions (communication, calculation, and information). It has service quality assurance for the processing, management, dissemination, and realization of synchronized information.

**Figure 5:**
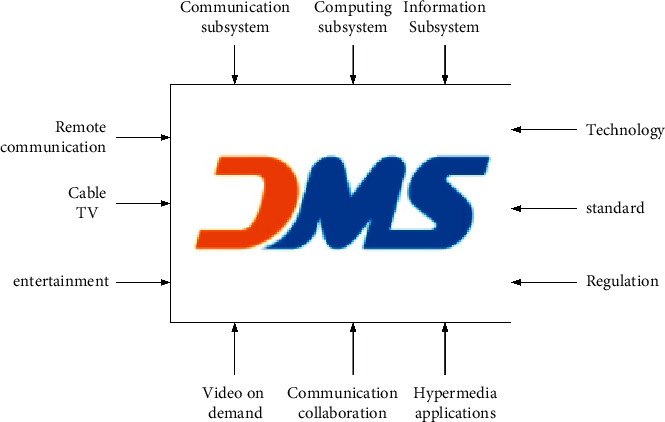
Distributed multimedia system.

Figure 5 shows the functions and applications of a distributed multimedia computer system. From a functional point of view, distributed multimedia expands the isolated multimedia system through a real-time network. It can provide services in interactive or broadcast modes and can also provide services in real time or in message transactions [22].

#### 2.3.2. Distributed Multimedia System Model

In order to meet the new requirements of distributed multimedia applications, the distributed multimedia system needs to provide necessary functions in the end system, where the client and the server are located and the communication network is connecting the client and the server at the same time.

**Figure 6:**
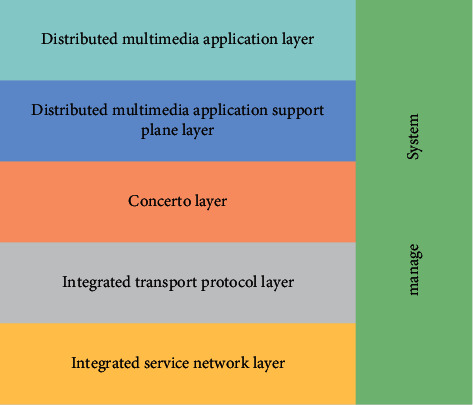
Distributed multimedia system model.

According to the research results at home and abroad about the own research experience, it is more appropriate for the distributed multimedia system to adopt the structural model shown in Figure 6.

*Integrated Service Network Layer*. This layer not only provides traditional data communication services, such as the front end of text file transmission, but also provides comprehensive services (multimedia communication services including continuous media, such as audio and video).

System management is performed at all levels of the reference model. If it is necessary to jointly complete the overall management of the system, corresponding management personnel need to be deployed in each layer, such as interlayer adjustment, peer adjustment, and so on. In a distributed multimedia system, system management not only provides conventional management services (e.g., configuration management, security management, accounting management, etc.), but also new requirements for multimedia applications, especially in order to meet the continuity requirements, need to provide management mechanisms (mainly to meet the new requirements of multimedia applications, especially the requirements of continuous media). This can be said to be one of the main problems that must be solved in the design and implementation of distributed multimedia systems.

## 3. Two-Way Cognitive Test Experiment

### 3.1. Two-Way Cognitive Algorithm Experiment

The data set used in the experiment is a subset of WordNet WN18 and a subset of FreeBase FB15k. WordNet is an English dictionary based on cognitive linguistics designed by Princeton University and supports automatic text analysis, including descriptions of attributes, components, and functions. FreeBase is a shared data set similar to Wikipedia. It is created by users themselves and stored in a graph format. Nodes are defined as entities, edges are defined as relationships, and each node and edge is assigned an id. The data set situation is shown in Table 1.

This article conducted experiments on the WN18 data set and the FB15K data set and conducted comparative experiments on each data set, namely, BP neural network-based method (BPNN) and weight adjustment method (WA) and BP neural network combined method; the specific statistics are shown in Tables 2 and 3.

The results of the experiment on the two data sets are not much different, which is reasonable. Due to the large scale of the data set in the experiment, the unevenness of the experimental data and the deviation of the experimental results caused by individual cases are not obvious.

However, the reasoning effect of the BP neural network algorithm is better. On FB15K, when the content of contradictory information is greater than 20%, the MAPE starts to rise. The reason is that the higher the content of contradictions, the greater the proportion of entity words, which leads to the BP neural network algorithm. The performance began to decline. It is obvious that combining the entity weight adjustment method with the BP neural network algorithm can overcome the abovementioned problems. It is worth noting that when the content of contradictory information is 0, there is still a MAPE value. However, in the experiment, the content of contradictory information is not set to be less than 5%, which shows that there is a small part of contradictory information in the data set itself because in the objective world, there is no noncontradictory state. But it is not difficult to see that when the content of contradictory information is very low, the BPNN + WA method can still improve the accuracy of knowledge reasoning. The statistics of the average percentage error of knowledge reasoning are shown in Table 4.

## 4. Simulation Analysis of Cognitive Computing Process

### 4.1. Cognitive Analysis of Information Transmission among Multiple People with the Same Thinking Mode

People with the same or similar thinking patterns have little difference in the way they think about problems. The information transmission between multiple people is a sample formed by converting the original concept into a random reality element of the concept and submitting the sample to the knowledge or acceptance of the concept. In order to make it identify the sample, after many cycles, the transmission of information is formed.

In the process of simulating the model, the original concept feature value is transformed into a sample set by forward CT, and then the inverse cloud algorithm is used. The sample is restored to the estimated value of the concept digital feature, and the process is repeated 100 times, which can be considered concept transfer information among 100 people. The specific method is as follows: the experiment simulates the transfer process of information among 100 people; that is, the same method is used to cycle cognition one hundred times. In this cycle, the number of cognition per person is set to 20 times, that is, starting from the first cognitive link. 20 samples are randomly generated, each sample contains 10,000 elements, and then the average of the 20 cognitive results is calculated as the final single cognitive result, and this result is used as the initial parameter for the next cognition. In this process, three kinds of reverse cloud computing algorithms are used to regulate the parameters of the samples. The specific parameters are shown in Table 5.

**Figure 7:**
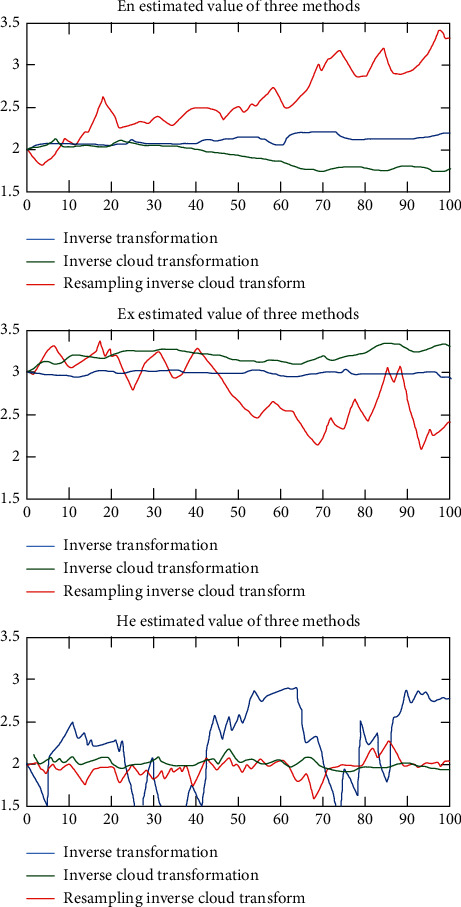
Cognitive calculation results of information transfer between multiple people.

The experimental results are shown in Figure 7.

According to the data trend in the figure, it can be seen that the increase in the number of transfers in the cognitive process between different people will lead to a decrease in the accuracy of the concept. This is due to the error amplification caused by the use of the previous expected value as the next parameter in the process of forward and backward CT, which is in line with the phenomenon of increased error in the process of information transmission between humans. From the perspective of the overall trend of expected value, the error of inverse CT and multistep inverse CT is small, and the error of resampling multistep inverse CT is greater than the above two methods. This is mainly due to the fact that the number of effective elements has not increased due to the random sampling in the sampling process of the multistep reverse cloud algorithm with repeatable sampling. But the overall accuracy is within the error range. From the perspective of all test results, when this cognitive method is used to process data, the difference in cognitive methods is mainly reflected in the cloud.

**Figure 8:**
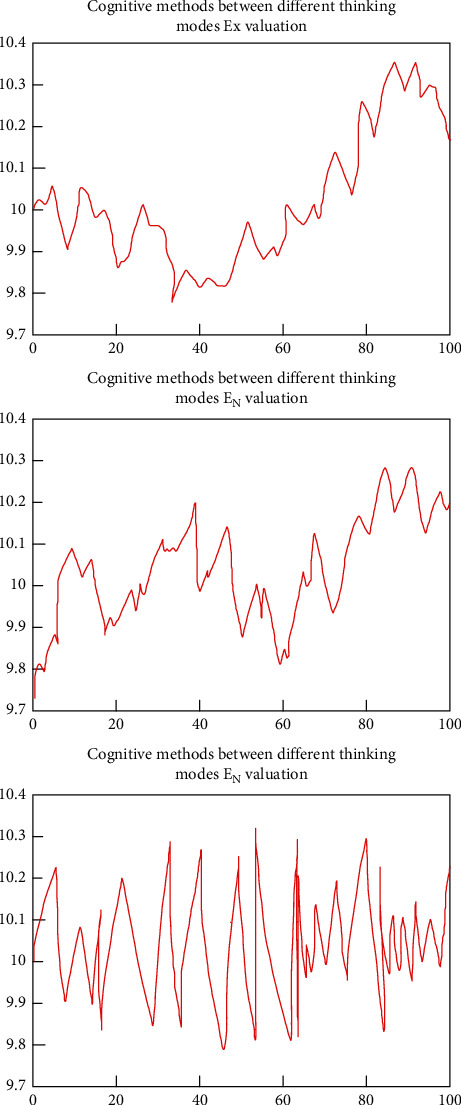
Cognitive transfer results between different thinking modes.

In daily life, people's thinking styles are not the same. Therefore, the transmission of information between people with different thinking modes will have greater variations, such as the communication between doctors and patients, the communication between doctors and doctors, and the news. Regarding the dissemination of events or the sharing of topics in the circle of friends, different people have different understandings of concepts. For the study of this model, the cognitive transfer between different inverse CTs can describe the phenomenon. To simulate the process, set up the experiment as follows: given the parameter value of the mathematical characteristic of the concept connotation (10, 2, 0.1), (20, 2, 0.4), the two sets of parameters are recognized 90 times in forward and reverse directions according to different methods and 20 times in a single recognition, and the average value is calculated, and the estimated value obtained is used as the input parameter for the next calculation. Thus, the results of the experiment are obtained. Comparing the results of the experiment to analyze, the specific results are shown in Figure 8.

It can be seen from the result of the above figure that the expected value Ex and the entropy En change basically the same and change within a certain range, and the accuracy rate is also higher. However, He has a large range of fluctuations, and the difference in cognitive processes between different individuals is mainly reflected in the hyper-entropy He. The more different ways of thinking, the more obvious this kind of performance.

### 4.2. Simulation Result Analysis

**Figure 9:**
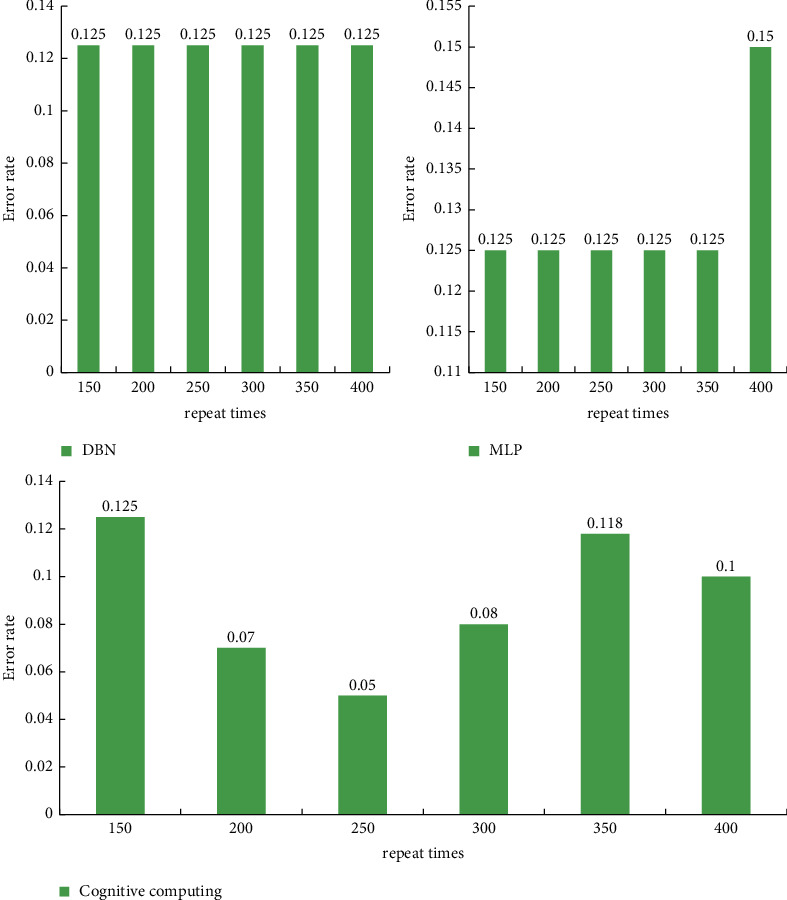
Comparison of the impact of the number of repetitions on the performance of three different algorithms.

In this experiment, the number of neurons in the hidden layer is controlled to 40, and the number of layers of the network is 3, and the number of repetitions of the training set is increased from the original 150 to 400, each time increasing by 50. The result is shown in Figure 9.

It can be obtained by analyzing the data that the accuracy of the deep belief network and the multilayer perceptron are basically the same after training with the same parameters. The multimedia-based cognitive computing calculation method proposed in this paper shows better accuracy than deep belief networks and multilayer perceptron when the parameters are selected reasonably.

**Figure 10:**
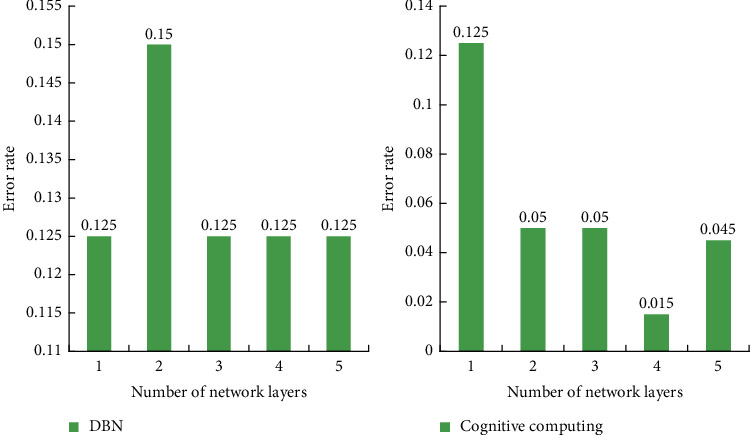
Comparison of the impact of the number of network layers on the performance of two different algorithms.

Modify the parameter of the number of network layers in increments of 1 from 1 to 5. The average value of each experiment was repeated 10 times. The parameter of the number of network layers is increased from 1 to 5 at intervals of 1, and each experiment is repeated 10 times to find the average value. The final corresponding result is shown in Figure 10.

From the experimental results, we can see that under the same number of network layers, the accuracy of the algorithm proposed in this paper almost completely exceeds the traditional deep belief network. According to the cross-check result, under this parameter, the theoretical decision of the algorithm in this paper is accurate to over 99.5%. Compared with the past, it has increased by 32.9%, effectively improving the performance of cognitive computing methods.

Based on the above analysis, we can see that the accuracy of the cognitive computing method after the rush has increased by 32.9%, and compared with the past, the cognitive model is more intelligent and can be well applied to the actual use process.

## 5. Conclusions

This article mainly studies the application research and improvement of cognitive computing methods. Through the use of computer-based multimedia technology, and the improvement of its cognitive computing method, it is improved to a two-way cognitive computing method, and at the same time, multimedia technology research is also carried out. After a comprehensive analysis, it is decided to use distributed multimedia technology, which has better security and faster speed and can be better applied to the topics studied in this article. And in the experimental part, some cognitive abilities are explored. In the analysis part, it compares and analyzes with the previous centralized mode and draws its advantages.

## Figures and Tables

**Table 1:** Data set statistics.

Data set	FB15K	WN18
Entity #E	38696	40942
Relationship #E	2478	18
Training set	272121	141443
Test set	20466	5000
Validation set	17535	5000

**Table 2:** Average absolute error of FB15K data set.

Method	BPNN (%)	BPNN + WA (%)
0	54	35
5	41	27
10	33	23
15	25	18
20	21	15
25	22	13
30	24	11

**Table 3:** Average absolute error of WN18 data set.

Method	BPNN (%)	BPNN + WA (%)
0	47	35
5	37	26
10	31	21
15	22	17
20	17	14
25	16	13
30	15	11

**Table 4:** The average absolute percentage error of knowledge reasoning.

Data set	FB15K		WN18	
	BPNN	BPNN + WA	BPNN	BPNN + WA
0	52.83	34.41	46.58	34.77
5%	39.54	27.12	37.29	26.12
10%	31.89	22.07	30.24	20.58
15%	24.7	17.48	21.65	16.99
20%	21.07	13.65	17.82	13.59
25%	21.82	12.4	16.57	12.44
30%	23.31	10.89	15.06	11.05

**Table 5:** Parameters' table.

Parameter name	Parameter value	
Ex	10	20
En	2	2
He	0.1	0.4

## Data Availability

The author does not have permission to share data from the data provider.
